# Differences in the distribution of HER2-positive breast tumors according to ethnicity and genetic variants in *ERBB2*: a special focus on Asian and Latina women

**DOI:** 10.3389/fonc.2025.1635681

**Published:** 2025-07-24

**Authors:** Laura Rey-Vargas, Lina María Bejarano-Rivera, Patricia López-Correa, Diego Felipe Ballen-Lozano, Silvia J. Serrano-Gómez

**Affiliations:** ^1^ Cancer Biology Research Group, National Cancer Institute, Bogotá, Colombia; ^2^ Doctoral Program in Biological Sciences, Pontificia Universidad Javeriana, Bogotá, Colombia; ^3^ Department of Pathology, National Cancer Institute, Bogotá, Colombia; ^4^ Clinical Oncology Unit, National Cancer Institute, Bogotá, Colombia; ^5^ Research Support and Follow-Up Group, National Cancer Institute, Bogotá, Colombia

**Keywords:** breast neoplasms, HER2 gene, American Native Continental Ancestry, single nucleoside polymorphism, ethnic group

## Abstract

**Background:**

HER2-positive breast tumors are clinically important breast cancer subtypes with an overall unfavorable prognosis, but also with current optimal treatment options that have significantly improved the patients’ survival. Several epidemiological registries have reported varying prevalence rates of HER2-positive breast tumors among population groups. In this review, we describe the prevalence of HER2-positive breast tumors by ethnicity, with a special focus on Asian and Latina women, along with genetic variants located in or near *ERBB2* that might affect its protein expression.

**Methods:**

We conducted a literature search for studies reporting differences in HER2-positive breast tumor prevalence among populations and HER2/*ERBB2* molecular features based on genomic background or ancestry.

**Results:**

Overall, Asian and Latina women tend to have higher proportions of HER2-amplified tumors, compared to non-Hispanic white (NHW) women. Additionally, higher Indigenous American ancestry is associated with an increased likelihood of HER2-positive tumors and elevated *ERBB2* expression. We also describe reported differences in the genotype of several genetic variants in *ERBB2* or nearby genomic regions according to HER2 expression, and mention variants in other genes that may also be associated.

**Conclusions:**

This literature review contributes to a better understanding of the underlying biology of HER2 expression in breast tumors, and the possible mechanisms that explain the differences in the distribution of HER2-positive subtypes among various population groups.

## 1 Introduction

Breast cancer is currently the most common malignancy diagnosed in women worldwide (46.8 per 100,000) and the leading cause of cancer mortality among women (12.6 per 100,000) (1). At the molecular level, breast cancer is a heterogeneous disease (2). Four major intrinsic subtypes have been described: luminal A, luminal B, human epidermal growth factor receptor 2 (HER2)-enriched, and basal-like. In the clinical setting, these subtypes can be identified by immunohistochemistry (IHC) techniques, mainly based on the expression of hormone receptors (HR) (estrogen (ER) and progesterone (PR) receptors) and the HER2 protein (3, 4). Each subtype has a different prognosis; patients with luminal A tumors have the best clinical outcome, whereas those with triple negative (TN) tumors or HER2 expression (whether classified as luminal/HER2+ or HER2-enriched subtypes), often present unfavorable outcomes as a consequence of these tumors’ aggressive phenotype (e.g., higher proliferation index and less differentiated tumors) (5). However, the clinical outcome of patients with HER2-positive tumors have significantly improved over the past years with the use of anti-HER2 therapy based on monoclonal antibodies such as trastuzumab, and tyrosine kinase (TK) inhibitors such as lapatinib (5, 6).

The prevalence of breast tumors with HER2 overexpression in the overall population ranges between 15% - 20% (4, 7), nonetheless, these percentages may vary according to ethnicity. Population-based studies have reported a higher proportion of HER2-amplified tumors in Asian and Latina women compared to non-Hispanic white (NHW) women (8–12). These variations can be partly related to the differences in the presentation of several reproductive and lifestyle factors between these population groups (13–15). However, in the past few years, various studies have stated that genetic-related factors can also contribute to HER2 expression in breast tumors (16–18). It has been described that the presence of genetic variants, such as single nucleotide polymorphisms (SNPs) in *ERBB2*, might influence the expression and activity of the HER2 protein (18–20). It is possible that these genetic variations are population-specific events (i.e., genetic variants whose allele frequencies are significantly higher in one population compared to others) that could contribute in part to the differences in the prevalence of HER2-positive breast tumors among population groups (21). Due to the clinical implications of the HER2 expression in breast tumors (6, 22), the aim of this review was to describe the prevalence of HER2-positive breast tumors by ethnicity, along with genetic variants located at *ERBB2* or nearby regions, that might affect its protein expression, with a special focus on reports for Asian and Latina women. We expect that this review will contribute to a better understanding of the underlying biology of HER2 expression in breast tumors, and the possible mechanisms that could explain the differences in the distribution of HER2-positive subtypes among various population groups.

## 2 Material and methods

**Figure 1:**
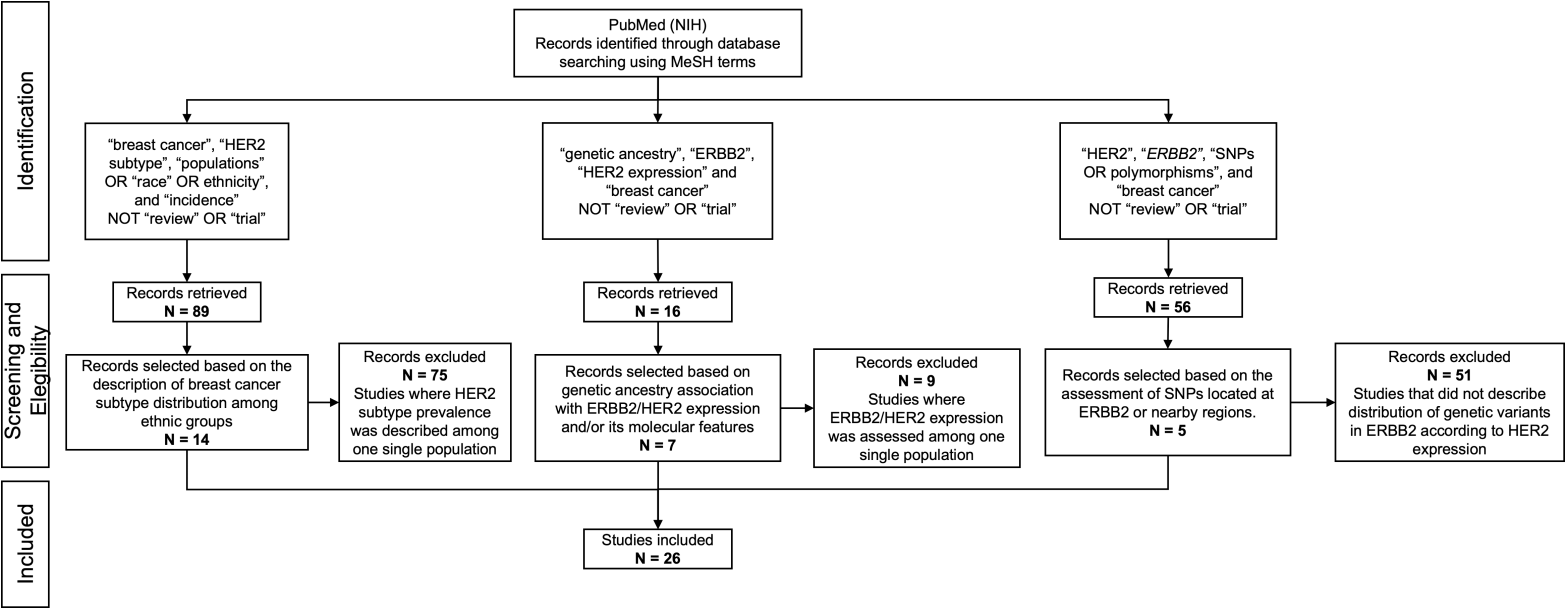
PRISMA flow diagram showing the database searches, the number of articles screened for eligibility, and the final number of full-text articles included.

The literature search was conducted in accordance with the Preferred Reporting Items for Systematic Reviews and Meta-Analyses (PRISMA) guidelines (**Figure 1**). The literature search was conducted using PubMed (NIH). We included original articles in English that assessed differences in HER2-positive breast tumors by ethnicity. This initial search included the Medical Subject Headings (MeSH) “breast cancer”, “HER2 subtype”, “populations” OR “race” OR ethnicity”, and “incidence”. To refine the search, the terms “review” and “trial” were excluded. 89 publications were retrieved from this search, and 14 articles that explicitly reported breast cancer subtype distribution among several ethnic groups were included. Studies where HER2 subtype prevalence was described among one single population were excluded as these articles do not allow comparisons among several groups. An additional search was conducted for articles that assessed genetic ancestry association with *ERBB2*/HER2 expression and/or its molecular features (amplification status, copy number variations, etc), using the MeSH words “genetic ancestry” “ERBB2”, “HER2 expression” and “breast cancer”, excluding the word “review”. A total of 16 publications were retrieved and 7 of these were included. Studies that evaluated the distribution of genetic variants or SNPs in *ERBB2* according to HER2 expression were also included. The MeSH words used were “HER2”, “*ERBB2”*, “SNPs OR polymorphisms”, and “breast cancer”; the terms “review” and “trial” were excluded. We focused mainly on SNPs located at *ERBB2* or nearby regions. 56 publications were retrieved, of which 5 were included. We did not limit the search by date. In total, 26 articles were selected for our review.

## 3 Results

### 3.1 Prevalence of HER2-positive subtypes (luminal/HER2 and HER2-enriched) by ethnic group

After the molecular characterization of breast tumors published by Perou et al. (23), differences in the distribution of intrinsic subtypes by ethnic groups have been widely reported (8, 24). It is well known that African American (AA) women are more likely to develop TN breast cancer, whereas NHW women have higher odds for luminal-like subtypes (25). Regarding HER2-positive tumors, its distribution among populations is less clear (**Tables 1**, **2**).

**Table 1:** Prevalence of HER2-positive breast tumors among different ethnic groups.

Reference	Sample size	Subtype	Biomarkers expression	NHWs	AAs	Asians	Latinas
SEER Cohort							
Akinyemiju et al. (26)	NHWs = 40,744 AAs = 6,007 Asians = 4,367 Latinas = 5,694	Luminal/HER2	ER+ and/or PR+, HER2+	9.7**%**	11.2**%**	15%	11.8**%**
		HER2-enriched	HR− and HER2+	4.0**%**	6.0**%**	5.4**%**	6.2**%**
Holowatyj et al. (27)	NHWs = 85,717 AAs = 10,540 Asians = 9,117 Latinas = 11,429	Luminal/HER2	HR+ and HER2+	11.2**%**	15.4**%**	14.0**%**	14.4**%**
CCR Cohort							
Clarke et al. (28)	NHWs = 50,248 AAs = 4,848 Asians = 8,441 Latinas = 12,700	Luminal/HER2	HR+/HER2+	9%	11%	12%	11%
		HER2-enriched	HR−/HER2+	4%	6%	8%	7%
Kurian et al. (10)	NHWs = 21,947 AAs = 2,071 Asians = 3,658 Latinas = 5,523	HER2-positive	HR+/HR- and HER2+	16.9**%**	22.4**%**	27.2**%**	24%
NCDB Cohort							
Sineshaw HM et al. (29)	NHWs = 126,856 AAs = 15,253 Asians = 5,121 Latinas = 8,299	Luminal/HER2	HR+/HER2+	8.9**%**	10.0**%**	10.6**%**	10.6**%**
		HER2-enriched	HR−/HER2+	3.8**%**	5.0**%**	6.1**%**	5.2**%**
LACE Cohort							
Kwan et al. (30)	NHWs = 1,943 AAs = 155 Asians = 189 Latinas = 197	Luminal/HER2	ER+ and/or PR+, HER2+	11.1**%**	9.0**%**	15.9**%**	14.3**%**
		HER2-enriched	HR− and HER2+	3.1**%**	3.2**%**	6.4**%**	6.6**%**
Sweeney et al. (8)	NHWs = 913 AAs = 115 Asians = 131 Latinas = 123	HER2-enriched	PAM50 classifier	12.5**%**	11.6**%**	12.4**%**	15.6**%**
AABCS, SFBCS and NC-BCFR Cohort							
John et al. (31)	NHWs = 273 AAs = 474 Asians = 1,106 Latinas = 941	HER2-enriched	HR−/HER2+	4%	17%	45%	33%
4 adjuvant chemotherapy trials Cohort							
Lipsyc-Sharf et al. (32)	NHWs = 7,889 AAs = 871 Latinas = 436 Non-Hispanic other: 283	HER2- positive	HR+/HR- and HER2+	47.3%	41.6%	59.1%	43.4%
4-Corners Breast Cancer StudyCohort							
Hines et al. (33)	NHWs = 119 Latinas = 69	HER2-positive	HR+/HR- and HER2+	14.3**%**	Not reported	Not reported	31.9**%**
Multi-institutional comparative analysis Cohort							
Nahleh et al. (34)	NHWs = 1,440 AAs = 233 Latinas = 1,566	HER2-enriched	HR− and HER2+	3.54**%**	5.45**%**	Not reported	6.32**%**
I-SPY 2 Cohort							
Kyalwazi et al. (35)	Whites (NHWs + Latinas) = 786 AA = 120 Asians = 68	Luminal/HER2	HR+/HER2+	17%	10%	15%	Grouped with NHWs
		HER2-enriched	HR−/HER2+	8%	11%	16%	

SEER, Surveillance, Epidemiology, and End Results; CCR, California Cancer Registry; NCDB, National Cancer Data Base; LACE, Life After Cancer Epidemiology; AABCS, Los Angeles County Asian American Breast Cancer Study; SFBCS, San Francisco Bay Area Breast Cancer Study; NC-BCFR, Northern California Breast Cancer Family Registry; I-SPY 2, Investigation of Serial Studies to Predict Your Therapeutic Response With Imaging and Molecular Analysis 2 trial; Adjuvant chemotherapy trials, CALGB 9741, CALGB 49907, CALGB 40101, and NCCTG N983; NHWs, non-Hispanic whites; AAs, African Americans; HR, hormone receptors; ER, estrogen receptor; PR, progesterone receptor.

**Table 2:** Differences in the odds for HER2-positive subtypes (luminal/HER2 or HER2-enriched) according to population groups.

Reference	Sample size	Subtype	Biomarkers expression	Odd ratio (95% CI)				Model
				NHWs	AAs	Asians	Latinas	
SEER Cohort								
Howlader et al. (36)	NHWs = 40,744 AAs = 6,007 Asians = 4,367 Latinas = 5,694	Luminal/HER2	HR+/HER2+	Reference	1.2 (1.0–1.3)	1.2 (1.1–1.4)	1.1 (1.0–1.2)	Model adjusted by age, stage, tumor grade, and SEER registry
		HER2-enriched	HR−/HER2+		1.4 (1.2–1.6)	1.8 (1.5–2.1)	1.4 (1.2–1.6)	
Holowatyj et al. (27)	NHWs = 85,717 AAs = 10,540 Asians = 9,117 Latinas = 11,429	Luminal/HER2	ER+/PR- and HER2+	Reference	1.14 (1.02–1.28)	1.03 (0.91–1.17)	1.04 (0.93–1.16)	Model adjusted for age, race, and poverty (quartiles)
CCR Cohort								
Parise et al. (37)	NHWs = 147,047 AAs = 13,847 Asians = 24,905 Latinas = 36.918	Luminal/HER2	HR+/HER2+	Reference	1.03 (1.01-1.20)	1.16 (1.09-1.23)	1.09 (1.03 - 1.15)	Model adjusted by stage, age, tumor grade, and socioeconomic status
Telli et al. (38)	NHWs = 60,498 AAs = 5,292 Asians = 9,113 Latinas = 14,106	HER2-positive	HR+/HR- and HER2+	Reference	1.2 (1.1-1.3)	Chinese: 1.1 (1.0-1.3) Philippine: 1.3 (1.2-1.5) Japanese: 1.0 (0.8-1.2) Korean: 1.8 (1.5-2.2) South Asian: 1.0 (0.8-1.3) Vietnamese: 1.13 (1.1-1.6)	1.1 (1.0 - 1.2)	Model adjusted for age at diagnosis, stage at diagnosis, tumor grade, neighborhood SES, year of diagnosis, nativity, and hospital ownership status
NCDB Cohort								
Sineshaw HM et al. (29)	NHWs = 126,856 AAs = 15,253 Asians = 5,121 Latinas = 8,299	Luminal/HER2	HR+/HER2+	Reference	1.13 (1.08–1.18)	1.07 (0.99–1.15)	1.11 (1.04–1.17)	Adjusted for diagnosis age, race, grade, stage, comorbidity, insurance status, census region and socioeconomic status
		HER2-enriched	HR−/HER2+	Reference	1.17 (1.10–1.25)	1.45 (1.31–1.61)	1.26 (1.16–1.37)	
LACE Cohort								
Kwan et al. (30)	NHWs = 1,943 AAs = 155 Asians = 189 Latinas = 197	HER2-enriched	HR−/HER2+	Reference	1.25 (0.49-3.21)	2.02 (1.05-3.88)	2.19 (1.16-4.13)	Adjusted for age at diagnosis, and Pathways/LACE study origin
Sweeney et al. (8)	NHWs = 913 AAs = 115 Asians = 131 Latinas = 123	HER2-enriched	PAM50 classifier	Reference	1.15 (0.55-2.38)	0.93 (0.55-1.54)	1.46 (0.71-3.01)	Adjusted for age at diagnosis
4-Corners Breast Cancer Study Cohort								
Hines et al. (33)	NHWs = 119 Latinas = 69	HER2-positive	HR+/HR- and HER2+	Reference	Not reported	Not reported	2.48 (1.10-5.58)	Adjusted for age and tumor characteristics
Multi-health institutional analysis Cohort								
Nahleh et al. (34)	NHWs = 1,440 AAs = 233 Latinas = 1,566	Luminal/HER2	HR+/HER2+	Reference	RRR = 1.03 (0.68, 1.56)	Not reported	RRR = 0.69 (0.55-0.87)	Adjusted by tumor characteristics
		HER2-enriched	HR−/HER2+	Reference	RRR = 1.46 (0.74-2.88)	Not reported	RRR = 1.61 (1.12-2.32)	
Hawai’i Pacific Health Tumor Registry Cohort								
Sasaki et al. (39)	NHWs = 979 Asians = 1,799 Filipino = 815 NHPI = 909	HER2-enriched	HR−/HER2+	Reference	Not reported	Premenopausal Asians: 1.13 (0.48-2.66) Filipino: 1.85 (0.76-4.48) NHPI: 1.31 (0.54-3.21) Postmenopausal Asians: 1.07 (0.63-1.79) Filipino: 1.66(0.95-2.89) NHPI: 0.79 (0.42-1.48)	Not reported	Premenopausal: Age at diagnosis, race, and year of diagnosis. Postmenopausal: age at diagnosis, race, histology, county, and year of diagnosis.

SEER, Surveillance, Epidemiology, and End Results; CCR, California Cancer Registry; NCDB, National Cancer Data Base; LACE, Life After Cancer Epidemiology; NHWs, non-Hispanic whites; AAs, African Americans; HR, hormone receptors; ER, estrogen receptor; PR, progesterone receptor; RRR, relative risk ratio; IRR, incidence rate ratio; IR, incidence rate; ALR, absolute lifetime risk CI, confidence interval.

#### 3.1.1 Overall trends from population and hospital-based registries

The Surveillance, Epidemiology, and End Results (SEER) registry, a large dataset that collected clinical and pathological information on cancer patients diagnosed in the United States (U.S) from 2010 to 2015, is estimated to cover approximately 97% of the incident cancers within the catchment area zones (40). In that sense, it gathers patients from different ethnic groups and represents a valuable resource to study breast cancer subtype distribution among several populations. SEER-based studies have consistently reported a higher prevalence (**Table 1**) and greater odds (**Table 2**) for HER2-positive subtypes in Asian, AA and Latina women. Akinyemiju et al. (26) showed a higher proportion of the luminal/HER2 subtype in Asian (15%), Latina (11.8%), and AA women (11.2%), compared to NHWs (9.7%). Moreover, specifically for the HER2-enriched subtype, they reported that Latinas presented the highest prevalence among all population groups (6.2%), followed by the AAs (6.0%), Asians (5.4%) and NHWs (4.0%). In the same way, Howlader et al. (36) reported a higher risk for HER2-positive subtypes in Latinas, either for the luminal/HER2 (odd ratio (OR)=1.1, 95% confidence interval (CI), 1.0–1.2) or the HER2-enriched subtype (OR=1.4, 95% CI, 1.2–1.6), compared to NHWs, in a model adjusted by age, stage, tumor grade, and SEER registry. Similar results were found in Asian and AA women for the luminal/HER2 (Asians: OR=1.2, 95% CI, 1.1–1.4; AAs: OR=1.2, 95% CI, 1.0–1.3) and the HER2-enriched subtypes (Asians: OR=1.8, 95% CI, 1.5–2.1; AAs: OR=1.4, 95% CI, 1.2–1.6). Furthermore, Holowatyj et al. (27) also reported a higher prevalence of the luminal/HER2 subtype in AAs (15.4%), Latinas (14.4%), and Asians (14.1%), compared with NHW women (11.2%), along with a statistically significant association between AA ethnicity and the luminal/HER2 subtype (OR=1.14, 95% CI, 1.02–1.28); they also found a tendency in Latinas and Asian women for having this subtype, using NHWs as the reference group (Latinas: OR=1.04, 95% CI, 0.93–1.16; Asians: OR= 1.03, 95% CI, 0.91–1.17).

Data from the National Cancer Data Base (NCDB) (29), a hospital-based but much larger U.S national cancer registry that covers approximately 70% of all newly diagnosed cancer cases in the country (41), included 260,174 breast cancer patients and consistently revealed that Latinas and AAs are more likely to develop luminal/HER2 tumors (Latinas: OR=1.11, 95% CI, 1.04-1.17; AAs: OR=1.13, 95% CI, 1.08–1.18) and the HER2-enriched subtype (Latinas: OR=1.26; 95% CI 1.16–1.37; AAs: OR=1.17, 95% CI, 1.10–1.25), compared to NHWs. This was also observed for Asian women, who presented the highest odds for HER2-enriched tumors among all populations (OR=1.45, 95% CI, 1.31–1.61). These findings in Asians were replicated by John et al. (31), in a cohort derived from three population-based studies—the Los Angeles County Asian American Breast Cancer Study (AABCS), the San Francisco Bay Area Breast Cancer Study (SFBCS), and the Northern California Breast Cancer Family Registry (NC-BCFR). Their analysis revealed that the highest prevalence of HER2-enriched tumors was observed in Asian women (45%), followed by Latinas (33%), whereas the prevalence among NHWs was reported at only 4%.

Reports on differences in HER2-positive breast tumors among ethnic groups have also been published using U.S state-wide cancer-based studies, like the California Cancer Registry (CCR), which gathers high quality data from all cancer patients diagnosed in 48 of California’s 58 counties. Clarke et al. (28) found a higher proportion of HER2-positive subtypes (either HER2-enriched or luminal/HER2) in Asians, Latinas, and AAs compared to NHWs (20%, 18% and 17% vs. 13%, respectively). In the same way, Telli et al. (38) also reported a higher likelihood of having either luminal/HER2 or HER2-enriched breast tumors in AAs and Latina women from the CCR (AAs: OR= 1.2, 95% CI, 1.1-1.3; Latinas: OR= 1.1, 95% CI, 1.0-1.2). Correspondingly, Parise et al. (37) reported higher odds for luminal/HER2 tumors in Asian and Latina women compared to NHWs, after adjusting for stage, age, tumor grade, and socioeconomic status (Asians: OR=1.16, 95% CI, 1.09-1.23; Latinas: OR=1.09, 95% CI, 1.03-1.15).

These results in Asians, AAs and Latinas have even been replicated in women from different parts of the U.S. Kwan et al. (30) conducted a study in 2,544 women from the Life After Cancer Epidemiology (LACE) study, which includes patients from the Kaiser Permanente Northern California Cancer Registry (KPNCAL) and the Utah cancer registry (UCR). They reported that Asian and Latina women had the highest prevalence of luminal/HER2 (Asian: 15.9%, Latina: 14.3%, NHWs: 11.1%, AAs: 9.0%; *p*=0.18) and HER2-enriched subtypes (Asian: 6.4%, Latina: 6.6%, NHWs: 3.1%, AAs: 3.2%; *p*=0.03) among all population groups; and also presented the highest odds for HER2-positive tumors (Asians: OR=2.02, 95% CI, 1.05-3.88; Latinas: OR=2.19, 95% CI, 1.16-4.16).

These findings consistently demonstrate a clear association between HER2-positive tumors and AA, Asian, and Latina ethnicities, suggesting the presence of disparities in the distribution of breast cancer subtypes among minority groups in the U.S. However, it is important to note that breast tumor subtype classification is often conducted based on the IHC expression of ER, PR and HER2 biomarkers (42). This approach is subjected to multiple limitations such as the variability of the staining and scoring by the pathologist, and cut-off points to define positive or negative cases, particularly for ER and PR (43). In contrast, gene expression-based assays account for a better approach for subtype classification. A study conducted by Sweeney et al. (8) based on the LACE registry described the overall distribution of breast cancer subtypes in relation to clinicopathologic categories among 1,319 women from different ethnic groups, applying the PAM50 classifier. The results showed that Latinas tend to present with non-luminal A subtypes, including HER2-enriched tumors (OR=1.46, 95% CI, 0.71–3.01), compared to NHWs. The same tendency was observed for AAs (OR=1.15, 95% CI, 0.55-2.38) but not for the Asian group (OR=0.93, 95% CI, 0.55-1.54), which may be explained by the small sample size of this group in the study and/or differences in the method used to define breast cancer subtypes. Gene expression-based technologies, such as PAM50, reflect in a better way the tumors biology, therefore, it is still necessary to keep exploring differences in breast cancer subtype classification among ethnic groups using molecular classifiers.

Most of the population-based research presented above includes large datasets of Caucasian patients, while minority groups such as Asians and Latinas are consistently underrepresented. This poses significant challenges for epidemiological analyses, particularly in terms of statistical power and the ability to draw conclusions that accurately reflect the realities of these populations. The same issue extends to genomic research, where limited diversity can obscure population-specific genetic variants (44). Therefore, improving the inclusion of these groups is essential to identify relevant biomarkers, refine informed therapeutic strategies, and ultimately reduce health disparities.

#### 3.1.2 Focused reports on the distribution of HER2-positive breast cancer in Asian and Latina women

In light of these findings, particular attention has been given to the Asian ethnic group to further investigate disparities among its subpopulations. For instance, Parise et al. (37) specifically focused on Asian ethnicities and reported a strong association between HER2-positive tumors and the Korean self-reported population (OR = 1.63, 95% CI: 1.38–1.99). Similarly, another CCR-based study who paid special attention to Asian also showed that of all population groups, Koreans present the highest prevalence (36%) and odds for HER2-overexpressing tumors (OR= 1.8, 95% CI, 1.5-2.2), followed by Philippines (OR= 1.3, 95% CI, 1.2-1.5) and Vietnamese (OR= 1.3, 95% CI, 1.1-1.6) (38). Comparable results were reported by Sasaki et al. (39) for premenopausal breast cancer patients across different Asian ethnicities, with ORs of 1.13 (95% CI: 0.48–2.66) for Asians, 1.85 (95% CI: 0.76–4.48) for Filipinos, and 1.31 (95% CI: 0.54–3.21) for Native Hawaiian or Pacific Islander women. These findings underscore the importance of disaggregated analyses among Asian subpopulations to identify significant variations in the prevalence and odds of HER2-positive breast cancer, differences that may otherwise be overlooked when these groups are studied collectively.

However, some reports fail to account for the significant heterogeneity within Asian subpopulations, leading to analyses that combine Asians with other ethnic groups. This was the case for a large study based on four adjuvant chemotherapy trials (CALGB 9741, CALGB 49907, CALGB 40101, and NCCTG N9831), which included 9,479 breast cancer patients (32). Among this population, only 2.98% corresponded to Asians and other ethnicities, such as American Indian or Alaska Native, Native Hawaiian, or Other Pacific Islanders. As a result, these women were grouped and analyzed collectively as “non-Hispanic other”. In this category, the prevalence of HER2-positive subtypes was notably higher (59%) compared to NHWs (47.3%), AAs (41.6%), and Latinas (43.4%), leaving unclear the potential disparities that might exist within the ethnicities included in the “non-Hispanic other” group (**Table 1**).

Grouping multiple ethnic populations into a single broad category results in a heterogeneous cohort. While this approach may increase the overall sample size and facilitate statistical analysis, it limits the ability to uncover meaningful differences or disparities that may exist among the distinct ethnic subgroups included within the aggregated category.

A similar scenario was observed for Latinas in a recent study based on the Investigation of Serial Studies to Predict Your Therapeutic Response with Imaging and Molecular Analysis 2 (I-SPY 2) (35). The study included 990 breast cancer patients from various self-identified ethnicities across different U.S. territories. However, due to the low representation of Latina patients (12% of the cohort), they were primarily grouped with the White women cohort. As a result, the distribution of the luminal/HER2 subtype revealed a higher prevalence among the White cohort (17%) compared to the Asian (15%) and African American (10%) cohorts. This highlights the challenges in drawing conclusions and comparing results across studies due to the mixed classification of patients within ethnic groups. It also reinforces the ongoing issue of minority underrepresentation in epidemiological research.

Although there is still an issue of underrepresentation of Latinas in the SEER and other population- and hospital-based studies (45, 46), recent efforts reflect a trend toward creating more representative population registries, with a particular focus on minorities. For instance, Nahleh et al. (34) conducted a multi-health institutional analysis where 45.5% (1,566/3,441) of the cases were U.S Latinas from Texas and California; other populations groups included were NHWs (1,440: 41.8%) and AAs (233: 6.7%). They evaluated differences in tumor characteristics among ethnic groups and found that, compared to NHWs, Latinas have a higher relative risk ratio (RRR) for HER2-enriched tumors (RRR=1.61, 95% CI, 1.12-2.32, *p=*0.01) after having adjusted by age at diagnosis, histological subtype, chemotherapy, and surgery. Interestingly, the association between luminal/HER2 tumors and Latin ethnicity was in the oppositive direction (RRR=0.69, 95% CI, 0.55-0.87, *p=*0.001), which might be related to the protective association for luminal breast tumors found in the Latinas group, compared to the NHW women (RRR= 0.76, 95% CI, 0.64-0.9, *p=*0.002). On the other hand, no statistically significant associations were found in AA breast cancer patients for neither of the HER2-overexpressing tumors (luminal/HER2 = 1.03, 95% CI, 0.68-1.56; *p*= 0.862HER2-enriched=1.46, 95% CI, 0.74-2.88, *p*= 0.273).

Specifically for Latinas, it is important to highlight that often in these epidemiological studies the prevalence of HER2-positive subtypes might be underestimated, given that HER2 status is less likely to be tested in this population group (47), and a higher proportion of these cases are excluded due to the lack of complete information. Even with these limitations, studies with small sample sizes have found similar results. For instance, a 4-Corners Breast Cancer-based study that only included 285 women (NHWs=119 and Latinas=69) reported a higher prevalence of HER2-positive tumors in Latinas compared to NHWs (31.9% vs 14.3%, *p*<0.001, respectively), and a higher likelihood of having HER2-positive tumors for this population group (OR=2.48, 95% CI, 1.10-5.58) in a model adjusted for age and tumor characteristics (33).

Other factors related to lifestyle (such as diet, exercise, alcohol consumption, tobacco use, among others) and reproductive behaviors (including contraceptive use, parity, and breastfeeding) have been explored in the search to elucidate potential contributors to health disparities in breast cancer (48, 49) as well as for each intrinsic subtype (50, 51), and are describe extensively elsewhere. Among these, in relation to HER2-positive tumors, it has been shown that having a family history of cancer, higher breast density, and obesity (body mass index >30) increases the odds of having this particular subtype (51). As these risk factors vary by ethnic group, it is possible that differences in their prevalence among populations may help explain the variation in the distribution of HER2-positive tumors described above (52).

Other reports have focused not only on the expression of HER2 but on the molecular features of its codifying gene, *ERBB2*, and its relationship with biological contributors that help explain differences in its distribution among ethnic groups. Therefore, we provide a description of the studies so far that have assessed *ERBB2* by ethnicity, and also, by genetic ancestry, as a more accurate definition of people’s ancestral origin.

### 3.2 Molecular features of *ERBB2* by ethnicity and genetic ancestry

Few studies have reported differences in *ERBB2* gene expression and its molecular features by population groups. Kan et al. (53) conducted a study to evaluate molecular differences between an Asian cohort of women from Korea (n=187) with breast cancer, and a group of Caucasians (n=745) and AAs (n=158) from The Cancer Genome Atlas (TCGA) database, and showed a higher frequency of somatic alterations (mutations and copy number variations (CNVs)) in *ERBB2* in the Asian cohort (20% vs. 9.1%, respectively), along with a higher proportion of HER2-enriched (8% vs 2.9%, respectively) and luminal/HER2 subtypes (14.4% vs. 10.4%, respectively) in Asian women, compared to the TCGA group (Caucasians + AAs). Similarly, Pan et al. (12) reported a higher level of amplifications in the *ERBB2* region (17q12) in a cohort of 560 Asian women from Malaysia, along with a higher prevalence of the HER2-enriched molecular subtype assessed by PAM50 in Asian women compared to a group of Caucasians from the TCGA (23.3% vs 9.9%, respectively).

In terms of gene expression, Grunda et al. (54) reported a higher expression of the *ERBB2* gene in Caucasians when compared to AA breast cancer patients (*ERBB2* mean fold change (FC) in Caucasians: 1.61 vs AAs: 0.63, *p*=0.012), and other genes associated with prognosis (*ESR1* and *GATA3*), disease progression (*HSPB1* and *SERPINA3*) and response to chemotherapy (*CLDN7* and *DLC1*). Even though the epidemiological studies described above usually report higher prevalence of HER2-positive tumors among AAs compared to NHWs, it is possible that Grunda’s et al. (54) conflicting results are related to the small sample size of the study (AAs= 11, Caucasians=11). It is also worth mentioning that the later study only assessed gene expression, whereas prevalence studies reported above are based on HER2 protein expression. Therefore, it is possible that several post-transcriptional mechanisms might be exerting an effect on *ERBB2*/HER2 expression, this way contributing to the reported differences between these population groups (55, 56).

Self-reported ethnicity comprehends a heterogeneous group with mixed ancestries (57). This has drawn attention to the importance of analyzing breast cancer molecular features by genomic estimated race or genetic ancestry. This estimation is based on the analysis of ancestry-informative markers (AIMs), which capture differences in allele frequencies across major continental populations. This approach allows ancestry to be quantified as an objective and continuous variable, enabling researchers to assess how varying proportions of European, Indigenous American (IA), African, and Asian ancestry may correlate with disease phenotypes (44).

This approach was applied in a recent study by Miyashita et al. (58), which assessed the genetic ancestry of 3,433 breast cancer patients from the Tempus Database. The study differentiated African ancestry patients (>20% African, <10% IA ancestry, and a combined African plus European likelihood >70%) from European ancestry patients (>80% European and <10% IA ancestry). Interestingly, the study found a higher enrichment of several hallmark and oncogenic gene signature sets in the European cohort compared to African ancestry patients, including the *ERBB2* gene set ERBB2_UP.V1_UP, which refers to a group of genes defined in the Gene Set Enrichment Analysis (GSEA) database that are consistently upregulated in the context of *ERBB2* pathway activation. However, this finding was only observed in stage IV tumors with an HR+/HER2- subtype. Although the enrichment of this gene set does not necessarily indicate that the *ERBB2* gene itself is expressed, it reflects the upregulation of genes associated with *ERBB2* activation and signaling. In this context, these findings strongly suggest the presence of important differences in the tumor biology of breast cancer patients, potentially driven by their genetic ancestry.

A study conducted in Latinas from Colombia by Serrano-Gomez et al. (16) assessed the association between genetic ancestry and gene expression in 42 women with luminal breast tumors. When patients were stratified according to the average fraction of IA ancestry (low IA <36%, high IA ≥ 36%), the high IA ancestry group showed a higher expression of *ERBB2*, along with other genes located at the HER2 amplicon, such as *GRB7* and *MIEN1*. In line with these results, Marker et al. (21) reported an association between the HER2-enriched subtype and the IA ancestry fraction in a cohort of 1,312 Peruvian women with breast cancer. They reported that the odds of presenting a HER2-enriched subtype increased by a factor of 1.30 per every 10% increase in IA ancestry (*p*=0.004) after adjusting for age at diagnosis, African ancestry fraction and height. This association was replicated in other sets of patients from Mexico (OR =1.20; 95% CI, 0.90–1.59) and Colombia (OR = 1.28; 95% CI, 1.03–1.60). In the same way, a separate study in Colombian women that included breast cancer patients from different health institutions around the country also reported a suggestive association between the IA ancestry and both HER2-enriched (OR =1.18; 95% CI, 0.50 - 2.67) and luminal B/HER2 subtypes (OR =1.61; 95% CI, 0.84 - 3.09), after adjusting for health institution, age at diagnosis, and clinical stage (59). This positions genetic ancestry as a potentially valuable tool in the clinical setting, as women with a higher contribution of IA ancestry may exhibit more frequent *ERBB2* overexpression, potentially making them more responsive to HER2-low targeted therapies, such as antibody-drug conjugates (ADCs) like T-DM1 and T-DXd.

Even though only a few studies have assessed *ERBB2* differential expression between population groups, the results gathered so far suggest that there are differences in gene expression by genetic ancestry that may account for differences in the biology, prognosis, and outcomes of breast cancer between population groups (52). For instance, epidemiological studies comparing population groups have reported that Latina women are more likely to present clinical characteristics associated with poor prognosis, such as being diagnosed at advanced stages (60). In line with this, a higher risk of breast cancer mortality has also been reported for Latina women compared to NHW women (61), which may ultimately be related to a higher frequency of more aggressive subtypes, like HER2-positive tumors. It is possible that variations in the distribution of the HER2-positive subtypes by ethnicity could be partly related to the presence of genetic variants.

### 3.3 HER2 overexpression according to genetic variants in *ERBB2*

Genetic variants are defined as specific changes in a genomic region that are partly responsible for phenotype differences reported between population groups, such as differences in susceptibility to various diseases, including cancer (62). Genetic variants or SNPs in *ERBB2* have been associated with breast cancer risk (63–65), therapy response and resistance to anti-HER2 treatments (66, 67). Given that most of the genetic variants reported so far in *ERBB2* are located at the transmembrane domain coding region, their main effect is related to changes in HER2 protein activity (68). In addition to this, other studies have found differences in HER2 expression according to the *ERBB2* SNPs genotype (**Table 3**).

**Table 3:** Differences in *ERBB2* SNPs genotype according to HER2-expression, and risk of HER2-positive tumors, among various population groups.

Reference	Population included	SNPs in *ERBB2*	Genotyped tissue	Position (GRCh38.p13)	Genotypic variation	Amino acid change	SNP classification	Genotype prevalence		Odd ratio (95% CI)	1KGP MAF
								HER2 positive	HER2 negative		
Su et al. (71)	303 women from China	rs2517956	Blood	chr17: 39,687,606	G>A	Not apply	UTR variant	G/A: 58.6% A/A: 26.3% G/G: 15.2%	G/A: 50.9% A/A: 17.5% G/G: 31.6%	Not reported	EUR: 0.681 AFR: 0.542 IA: 0.546 EA: 0.402 SA: 0.695
		rs1058808	Blood	chr17: 39,727,784	C>G	Pro>Ala	Missense variant	C/G + G/G: 68.4% CC: 31.6%	C/G + G/G: 84.8% CC: 15.2%	Not reported	EUR: 0.673 AFR: 0.192 IA: 0.478 EA: 0.402 SA: 0.609
Cresti N et al. (17)	361 women from the United Kingdom	rs1058808	Blood and tumor	chr17: 39,727,784	C>G	Pro>Ala	Missense variant	C/G + C/C = 56% G/G: 43%	C/G + C/C: 44% G/G: 57%	Not reported	
Han W et al. (74)	90 women from South Korea	rs1058808	Blood	chr17: 39,727,784	C>G	Pro>Ala	Missense variant	Haplotype I (rs1058808: C; rs1136201: A) = 86.6% Other haplotypes = 13.4%	Haplotype I (rs1058808: C; rs1136201: A) = 80.6% Other haplotypes = 19.4%	Haplotype I: 1.5 (1.11-2.16)	
		rs1136201	Blood	chr17: 39,723,335	A>G	Iso>Val	Missense variant				EUR: 0.246 AFR: 0.010 IA: 0.137 EA: 0.124 SA: 0.131
Pivot X et al. (79)	8,703 women from France	rs68130068	Blood	chr2: 172285284	C>T	Not apply	Intronic region	C/C: 69.66% C/T: 27.52% T/T: 2.82%	C/C: 73.46% C/T: 24.73% T/T: 1.81%	T/T genotype: 1.88 (1.33-2.65) C/T genotype: 1.20 (1.07 - 1.34)	EUR: 0.146 AFR: 0.078 IA: 0.089 EA: 0.084 SA: 0.139

SNPs, single nucleotide polymorphism; Pro, proline; Ala, alanine; Iso, isoleucine; Val, valine; UTR, untranslated region; CI, confidence interval; 1KGP, 1000 Genomes Project Phase 3; MAF, minor allele frequency; EUR, European; AFR, African; IA, Indigenous American; EA, East Asian; SA, South Asian.

Su et al. (71) evaluated the expression of the HER2 protein according to the SNP rs1058808 genotype, a genetic variant (C>G) located at the *ERBB2* gene residue 1170 that encodes for either a proline (C allele) or alanine (G allele) at the C-terminal region of the HER2 protein (69). This study that included 303 Chinese women reported that patients with the C/G and G/G germline genotypes had a higher frequency of HER2-positive tumors, compared to patients with the C/C genotype (C/G: 58.6% and G/G: 26.3% vs. C/C: 15.2%, *p* = 0.007). This difference was also statistically significant under the dominant model (C/G + G/G: 84.8% vs. C/C: 15.2%, *p* = 0.003). Nonetheless, opposite results were reported in a European population, in a study that included 361 breast cancer patients from the United Kingdom. They analyzed germline and tumor genotype of numerous SNPs, including the rs1058808 and HER2 expression by IHC and found a significantly higher proportion of HER2 positive tumors in the proline carriers (either C/G or C/C), compared to the alanine carriers (G/G genotype) (56% vs. 43%, respectively, *p*=0.015) (17). These contradictory results might be partly explained by the differences in the rs1058808-G minor allele frequency (MAF) between both populations (1000Genomes project (1KGP): Asians G=0.4018 vs Europeans G=0.6730) (72), coupled with the flip-flop phenomenon, where the same allele confers risk in one population but is protective in another (73), although it is also possible that sample size differences between both studies might contribute as well to these conflicting results. On the other hand, these studies only assessed the differences in the proportion of HER2 positive cases according to rs1058808 genotype, therefore, it is likely that other biological factors, meaning additional genetic variants beyond rs1058808, might also contribute to the expression of the HER2 protein in these diverse populations.

The same genetic variant (rs1058808) was also evaluated in another study by Han W et al. (74) in 90 breast cancer women from South Korea. The association with HER2 expression was assessed for the rs1058808 SNP and at the same time, for five other SNPs at the *ERBB2* gene, all together as a haplotype. They reported that patients carrying the germline haplotype configuration I, which includes the rs1058808-C allele, were 1.5 times more likely to develop tumors with HER2 overexpression, compared to patients without this specific genotype (OR=1.5; 95% CI, 1.11-2.16, *p*= 0.009). This haplotype also included the rs1136201 SNP (I655V), a genetic variation (A>G) that leads to an isoleucine (I)-to-valine (V) substitution at codon 655 within the transmembrane domain of the *ERBB2* gene (63). This haplotype containing the rs1058808-C and rs1136201-A alleles, was found associated with HER2 overexpression among Korean breast cancer patients (74). According to the 1KGP, both SNPs alleles are considerably more frequent among East Asians, compared to other populations such as Europeans (East Asians: rs1058808-C=0.571 and rs1136201-A=0.8760 vs. Europeans: rs1058808-C= 0.33232and rs1136201-A=0.7545) (72, 75). In that sense, these SNPs genotypes might contribute to the higher prevalence of the HER2-positive tumors reported above among Asian populations when compared to Europeans. Several meta-analyses have reported an association between the rs1136201 genotype and breast cancer risk (63, 76), nonetheless, the impact of this genetic variation on the expression of its protein remains unclear.

Other types of genetic variants that localize at non-coding gene sequences have been reported to affect gene expression (70, 77). One of these genetic variants, the rs2517956, a 2 Kb upstream variant (G>A) of the *ERBB2* gene (78), was genotyped in 303 breast cancer patients from China, and evaluated according to the HER2 protein expression by IHC. This study found a higher proportion of HER2 positive tumors among patients with the A/A germline genotype, compared to homozygous G/G breast cancer patients (26.3% vs 15.2%, respectively, *p=*0.008) (1). However, the highest proportion of HER2-positive tumors was actually found among cases with the heterozygous A/G genotype (58.6%), suggesting this association might have an alternative underlying mechanism to the additive model. Additionally, it is worth noting that the overall rs2517956 allele’s frequency reported on the 1KGP (78) is relatively uniform across population, suggesting that this variant is unlikely to contribute to differences in HER2 expression in breast tumors among ethnic groups.

An important effort to elucidate potential genetic variants that confer risk for HER2-positive breast tumors was made by the French National Cancer Institute through a case-case Genome-Wide Association Study (GWAS) in over 8,703 women (79). They identified a SNP (rs68130068) located at chromosome 2 within an intronic region, which was potentially associated with HER2-positive breast tumors. Even though this SNP genotype (C>T) does not affect the sequence of any nearby gene, they reported that patients with the homozygous genotype for the minor allele (T/T) were 88% more likely to have HER2-positive breast tumors (OR=1.88; 95% CI, 1.33-2.65, *p*=0.00033), compared to patients with the C/C genotype. Heterozygous (C/T) patients also showed higher risk for HER2-positive tumors when compared to the C/C group (OR=1.20; 95% CI, 1.07-1.34, *p*=0.0013). Nonetheless, when the genome-wide association was tested, the rs68130068 SNP only reached a borderline level of significance (*p*=3.6x10^-6^).

Even though the mechanism by which the rs68130068 variant and others that are located at non-codifying genome sequences might contribute to the tumor phenotype is not entirely understood, it has been hypothesized that these SNPs regulate gene expression through several allele-specific mechanisms according to the distance from the regulated gene (80). SNPs that map close are referred to as cis- expression quantitative trait loci (eQTLs), whereas SNPs that map far from the regulated genes are referred to as trans-eQTLs (81). In that sense, it is possible that these eQTLs, whose allele frequencies vary across specific populations or ancestry groups, may influence the transcription of *ERBB2* or other genes upregulated in HER2-positive tumors. This could provide a potential biological mechanism underlying the disparities observed between populations for this particular breast cancer subtype.

Other studies have also reported differences in HER2 expression in breast tumors according to the genotype of SNPs located at other genes with roles related to gene regulation (*FOXP3*, *NOTCH3*) (82, 83), hormone signaling (*ESR1* and *CYP19A1*) (84–86) and proliferation/survival control (*WISP-1*, *CASP8*, *KRAS*, *TGFBR2*, *VEGF-A* and *CCND3*) (87–92). Possibly, the impact of these SNPs on the HER2 expression arises from their regulatory effects on the gene in which they are located, indirectly influencing the expression of other genes and proteins, such as *ERBB2*/HER2. In this context, differences in the genotype frequencies of these SNPs across populations may partially explain the disparities reported in HER2-positive breast tumors among ethnic groups. Further research is needed to deepen our understanding of health disparities between populations and to shed light on the underlying biology of HER2 expression in breast tumors.

## 4 Limitations and perspectives

This review certainly has some limitations, primarily related to topics that were not addressed in depth. For instance, while we reviewed differences in HER2-positive breast tumors across population groups, such differences may also influence patient outcomes and contribute to disparities in survival rates among these groups. However, these issues have already been thoroughly explored in other publications (52, 93). Similarly, although we describe studies that have analyzed genetic ancestry and its potential influence on the prevalence of HER2-positive tumors, the underlying genetic mechanisms are not discussed in detail, as this remains an area of ongoing investigation. Nevertheless, we hypothesize that this may be related to the presence of certain SNPs whose genotype frequencies vary across populations and have been associated with HER2 expression. Still, many other genetic variants, within different genes and genomic regions, may also be involved. As this is a broad and evolving topic, further systematic reviews are needed to consolidate and analyze the growing body of literature concerning the molecular epidemiology of HER2-positive breast cancer.

## 5 Conclusions

We reviewed studies that reported differences in HER2-positive breast cancer subtypes among population groups and consistently found a significantly higher prevalence of these tumors in Latina and Asian women. In some reports, this was also observed in AA women. Furthermore, studies that analyzed breast cancer patients according to genetic ancestry showed that a higher IA ancestry fraction is associated with *ERBB2*/HER2 expression. These results suggest that variations in the distribution of HER2-positive subtypes by ethnicity could be partly related to differences in allele frequencies of certain genetic variants among populations. We reviewed SNPs that can be found directly at the *ERBB2* gene sequence, either affecting the protein structure or its transcriptional regulation. However, other reports have described SNPs located at independent genes that, due to their biological functions, may also affect HER2 expression in an indirect manner. Further studies with larger sample sizes are still needed to elucidate if differences in some of the aforementioned SNPs genotypes can actually contribute to a higher risk of developing HER2-positive breast cancer.
